# Role of HCV infection in the development of carotid atherosclerosis in a cohort of HIV-infected patients

**DOI:** 10.1186/1758-2652-13-S4-P204

**Published:** 2010-11-08

**Authors:** S Ferrara, A Tartaglia, T Santantonio, B Grisorio

**Affiliations:** 1 Infetious Disease Unit., Ospedali Riuniti of Foggia, Foggia, Italy; 2 Clinic of Infectious Disease, University of Foggia, Foggia, Italy; 3 Infectious Disease Unit - Ospedali Riuniti of Foggia, viale L. Pinto, 1, Foggia, Italy

## Background

HIV-infected patients have an increased risk of cardiovascular disease. Measurement of carotid artery intima-media-thickness (c-IMT) with colour-doppler-ultrasonography is a well-accepted, non-invasive method to assess carotid atherosclerosis.

## Purpose of the study

To investigate whether HCV infection could be involved in the development of carotid atherosclerosis beside the well-known risk factors.

## Patients and methods

In this cohort study, 322 consecutive HIV+ subjects were identified and enrolled between May 2009 and May 2010. A total of 153 patients were HIV/HCV co-infected, whereas 169 were HIV+ mono-infected; 237 patients were treated with highly active antiretroviral therapy (HAART), and 85 subjects were HAART-naïve. All patients underwent at least one c-IMT measurement by the same examiner; an IMT of >0.9 mm was considered pathological.

## Results

Overall, 112/322 (35%) patients showed c-IMT >0.9. Table 1 shows the correlation between c-IMT and the following risk factors: age, cigarette smoking, intravenous drug use, CD4 cell count <200/mmc, CDC stage C of HIV infection, PI-based regimens and HCV co-infection. A significant statistical association between all considered factors and increased c-IMT was found. In particular, HCV co-infection showed a greater association in addition to older age, dyslipidemia, stage C of HIV infection.

**Table 1:** 

	c-IMT<0.9		c-IMT> 0.9		
	n. 210	%	n. 112	%	p

age >40 years	121	57.6%	105	93.8%	<0.0001

cigarette smoking	42	20%	70	63%	0.03

IVDU	79	37.6%	56	50%	0.03

Cholesterolemia >200 mg/dL	50	23.8%	51	45.5%	0.0006

Triglyceridemia >170 mg/dL	58	27.6%	52	46.4%	0.0006

CDC Stage C	69	38.9%	68	60.7%	<0.0001

HAART - PI exposure	56	40.9%	81	59.1%	0.001

CD4 cell count <200/mmc	36	17.1%	36	32.1%	0.002

HCV co-infection	85	40.5%	68	60.7%	0.0005

					

## Conclusions

In this cohort, several risk factors seem contribute to inflammatory damage and c-IMT development. Among them, HCV co-infection has been identified as a major determinant of carotid atherosclerosis. If the role of HCV infection will be confirmed in further studies, HIV-HCV co-infected patients should be strictly monitored for the vascular status.

